# Potential Autoepitope within the Extracellular Region of Contactin-Associated Protein-like 2 in Mice

**DOI:** 10.9734/BJMMR/2014/6135

**Published:** 2014-01-01

**Authors:** Demian F. Obregon, Yuyan Zhu, Antoinette R. Bailey, Samantha M. Portis, Huayan Hou, Jin Zeng, Saundra L. Stock, Tanya K. Murphy, Michael A. Bengtson, Jun Tan

**Affiliations:** 1 Rashid Laboratory for Developmental Neurobiology, Silver Child Development Center, Department of Psychiatry and Behavioral Neurosciences, Morsani College of Medicine, University of South Florida, MDT 14, 3515 East Fletcher Avenue, Tampa, Florida, 33613, USA; 2 Psychiatry and Behavioral Neurosciences, Rothman Center for Neuropsychiatry, Morsani College of Medicine, University of South Florida, 800 6^th^ Street South, Box 7523, Saint Petersburg, Florida, 33701, USA; 3 Department of Pediatrics, Rothman Center for Neuropsychiatry, Morsani College of Medicine, University of South Florida, 800 6^th^ Street South, Box 7523, Saint Petersburg, Florida, 33701, USA

**Keywords:** CNTNAP2, Caspr 2, autoantibody, molecular mimicry, autoimmune, autoepitope, autism, encephalopathy

## Abstract

**Aims:**

Implicated in autoimmune encephalitis, neuromyotonia and genetic forms of autism, here we report that contactin-associated protein-like 2 (CNTNAP2) contains a potential autoepitope within the extracellular region.

**Methodology:**

CNTNAP2 sequence-similar regions (CSSRs) from human pathogens were identified. Sera from autistic and control children were obtained and analyzed for the presence of antibodies able to bind CSSRs. One such candidate CSSR was evaluated for evidence of autoimmune responses to CNTNAP2 in a mouse model of acute infection.

**Results:**

Autistic and control children sera contained antibodies able to discrete regions of CNTNAP2. In a murine model of acute infection, a CSSR derived from the N-terminal extracellular region of CNTNAP2 resulted in anti-CNTNAP2 antibody production, proinflammatory cytokine elevation, cerebellar and cortical white matter T-cell infiltration as well as motor dysfunction.

**Conclusion:**

Taken together, these data suggest that CNTNAP2 contains a potential autoepitope within the extracellular region.

## 1. INTRODUCTION

Molecular mimicry is a process whereby an amino acid sequence-similar region, or shape-similar region, of a protein or other non-protein compound from an offending pathogen or other agent induces the production of adaptive immune system elements such as antibodies and/or T-cell receptors capable of cross-reacting with proteins or other compounds of the host. This pathological mechanism is thought to be involved in several conditions including: paraneoplastic cerebellar syndrome [1], autoimmune neuromyotonia [2], multiple sclerosis and related disorders [3,4], NMDAR encephalitis, and other autoimmune encephalopathies [5–7] as well as some developmental disorders including autism spectrum disorders [8–12]. Interestingly contactin-associated protein-like 2 (CNTNAP2), also known as Caspr2, is a member of the neurexin family and the target of autoantibodies thought to result in autoimmune encephalitis and neuromyotonia [2,13]. Furthermore, CNTNAP2 gene mutations are associated with autism [14–16]. The CNTNAP2 protein is expressed on neurons, neural stem cells, and astrocytes and is known to function in potassium channel clustering on myelinated axons, neuronal migration, membrane excitability, and neuron-glial interactions [17–19]. In early childhood, inflammatory insults may alter brain development as significant cross-over exists between molecular signaling pathways critical for brain development and those involved in immune responses [20–23]. Given its involvement in autoantibody-mediated neuroinflammatory disease, the investigations presented here evaluated CNTNAP2 for potential autoepitopes through a bioinformatics approach coupled with characterization of human CNTNAP2 binding antibodies from autistic and non-autistic children, and evaluation of a pathogen protein with similar linear protein sequences to human CNTNAP2 in a mouse model of acute infection.

## 2. METHODOLOGY

### 2.1 Cell Culture

Murine neuroblastoma cells (N2a) were purchased from the American Type Culture Collection (ATCC, Manassas, VA) and were cultured in Dulbecco’s minimum essential medium (DMEM, Life Technologies, Gaithersburg, MD) with 100 μg/mL penicillin and streptomycin antibiotics at 5% CO_2_ and 37°C. N2A cells were induced to differentiate by addition of 0.3 mM dibuturyl cAMP for 48–72 h. Cells were cultured in 24 or 96 well tissue culture for experimentation.

Mouse primary culture neuronal cells were isolated between E16 and E18 from cerebral cortices of mice subjected to LPS pre-treatment and CSSR3 peptide immunization. Cortices were incubated for 15 min in 0.25% trypsin at 37°C, and then mechanically dissociated. Single cells were collected after centrifugation at 290 x g and resuspended in DMEM supplemented with 10% fetal calf serum, 10% horse serum, uridine (33.4 μg/ml; Sigma) and fluorodeoxyuridine (13.6 μg/ml; Sigma). Cells were then plated in collagen-coated 24-well tissue culture plates at 2.5 × 10^5^ cells per well for experimentation.

### 2.2 Enzyme-Linked Immunosorbent Assays

CNTNAP2 sequence-similar regions (CSSR) were determined comparing human [NCBI Reference Sequence: NP_054860.1] and mouse [NCBI Reference Sequence: NP_001004357.2] CNTNAP2 proteins [24] against non-redundant protein sequences from available bacterial and viral protein databases using the Protein-Basic Local Alignment Search Tool (BLAST) [25] (Step 1). Resulting CSSRs (Table 1) at least 5 amino acids (aa) in length, within the predicted extracellular region of both human and murine CNTNAP2, were further analyzed by a battery of B-cell epitope prediction software tools [26–28] (Step 2). From those, only known human pathogens (bacterial or viral only) proteins were selected and then further analyzed based on linear protein sequence similarity with known B-cell epitopes [29] (Step 3). The final CSSRs from Step 3 were selected *a priori* and synthetic peptides (Table 2) were designed to contain a given CSSR flanked by sufficient amino-acids from human CNTNAP2 so as to generate peptides 8 amino acids in length.

A region of human CNTNAP2 at amino acids (aa) 41–49, not containing sequence similarity with the final CSSR peptides, was selected as a peptide control. Antibody titers were quantified using ELISA whereby individual CSSR peptides were first diluted to 1 μg/mL in 50 mM carbonate buffer (pH 9.6) and then used to coat 96-well plates at 100μL per well for 18 h at 4°C. Plates were next washed 5 times with phosphate buffered saline (PBS), 0.05% TWEEN-20, at pH 7.4 (wash buffer). Wells were blocked with 1% bovine serum albumin (BSA) and 5% horse serum in PBS for 2 h at room temperature. Following blocking, the plates were washed 5 times with wash buffer. Sera samples from autistic and control children (Table 3) were diluted (1:100) with 1% BSA in PBS.

Samples and standards were incubated in plate for 2 h at room temperature. After this incubation, the plates were washed 5 times with wash buffer, secondary antibody (anti-human IgG conjugated with HRP, produced in rabbit, 1:5000 dilution) incubation was conducted for 2 h at room temperature then 5 further washes with wash buffer and finally the plates were developed with tetramethylbenzidine substrate-chromogen (Dako, Carpinteria, CA, USA). The reaction was stopped with 2 N sulfuric acid and the plates were analyzed spectrophotometrically at 450 nm. Commercially available ELISA kits were used to measure tumor necrosis factor-α (TNFα; eBioscience, San Diego, CA) and interferon-γ (IFNγ; R&D Systems, Minneapolis, MN) levels in mouse sera and brain tissues. Experiments were performed according to manufacturers’ instructions.

### 2.3 Cytotoxicity Assay

Sera from individual mice were pooled together based on treatment group after isolation. Next 10 μL was diluted (1:100) in culture media and then incubated with N2a cells in 96 well plate for 24 h with and without 1 hour pre-incubation with CSSR3 or CNTNAP2 ctrl peptides (5 μg/mL). Media were then collected and analyzed for lactate dehydrogenase (LDH) release (Sigma) according to the manufacturer’s instructions.

### 2.4 Mouse Husbandry and Treatment

Wild-type C57BL/6 mice were purchased (Jackson Laboratories, Bar Harbor, ME) and housed in a 12-h light-dark cycle. Mice (4 week old, n = 8, 4♀/4♂ per group, 6 groups, total 54 mice) were treated via intraperitoneal (i.p.) injection with PBS or LPS (10 μg/mouse); and with and without immunization against (200 μg/mouse) synthetic peptides including pathogen peptide (NCBI Reference Sequence: NP_880571.1, filamentous hemagglutinin protein from *Bordetella pertussis*, 3034–3053 aa, sequence AGTSVDA**ANVSID**AGKDLNL) containing CSSR or Control peptide (NCBI Reference Sequence: NP_054860.1, CNTNAP2 31–50 aa, sequence TSQKCDEPLVSGLPHVAFSS); a portion of CNTNAP2 found not to have significant linear protein sequence similarity to known human bacterial or viral pathogen proteins. All treatments were repeated after one week.

For tissue collection, mice were anesthetized using gaseous isoflourane. Blood was collected from the right ventricle of the heart and immediately placed into tubes containing 0.5 M EDTA (BD Biosciences, San Jose, CA). Mice were transcardially perfused with cold 0.01 M PBS (pH 7.4) and brains were rapidly removed and sagittally bisected. Left hemispheres were separated into cerebrum and cerebellum regions before homogenization in 1 X lysis buffer (Cell Signaling, Boston, MA) with 1% PMSF (Sigma), centrifuged at 14,000 rpm for 15 min and stored at −80°C. Right hemispheres were fixed overnight with 4% paraformaldehyde and cryoprotected in a graded series of 10%, 20% and 30% sucrose solutions, each overnight at 4°C. Right hemispheres were then embedded in Neg50 frozen section medium (Richard-Allan Scientific, Kalamazoo, MI), and coronally sectioned on a Microm HM 550 cryostat (Thermo Scientific, Richard-Allan Scientific, Kalamazoo, MI) at 25 μm thickness. Free-floating sections were preserved in PBS containing 100 mM sodium azide at 4°C. All experiments and tissue collection were conducted in accordance with the institutional guidelines and were approved by the University of South Florida Institutional Animal Care and Use Committee.

### 2.5 Immunohistochemistry

Mouse brain tissue sections were washed in PBS, blocked in 5% horse serum/PBS for 1 h at room temperature and incubated with primary antibodies against CD3 or CD4 (rat, 1:1,000) overnight at 4°C in blocking solution. Sections were then washed and incubated for 1 h with biotinylated secondary antibody (anti-rat, 1:200) that was viewed by the ABC kit (Vector Laboratories) with diaminobenzidine (DAB). Slides were counterstained with hematoxylin. Images were obtained using an Olympus BX-51 microscope.

### 2.6 Motor Function Analysis

Motor coordination and balance were tested by placing each mouse on a rotating drum (RotaRod, UgoBasile, Stoelting, Wood Dale, IL) stationary and during acceleration from 0 to 40 rpms over a 5 minute period. Six mice per trial were randomly evaluated by a technician blinded to their identities. Each mouse was subjected to this task 5 times with a 30-min interval between each trial on RotaRod. All mice were tested on the same day.

### 2.7 Human Sera Samples

Sera from autistic (n = 26) and non-autistic children (n = 18) aged 3–11 years (Table 3) were obtained from the Autism Genetic Research Exchange (AGRE) [30]. Approval for study involving these specimens was granted by the institutional review boards of the University of South Florida, Morsani College of Medicine and AGRE. Autism diagnosis was determined using the Autism Diagnostic Interview - Revised (ADI-R) [31].

### 2.8 Statistical Analyses

All data were normally distributed; therefore, in instances of single mean comparisons, Levene’s test for equality of variances followed by t-test for independent samples was used to assess significance. In instances of multiple mean comparisons, analysis of variance (ANOVA) was used, followed by post-hoc comparison using Bonferonni’s method. Alpha levels were set at .05 for all analyses. The statistical package for the social sciences release 18 (SPSS, Chicago, IL) was used for all data analysis.

## 3. RESULTS AND DISCUSSION

### 3.1 CSSR of Proteins from Human Pathogens

Molecular mimicry is a well-known phenomenon known to underpin many disorders. To test the hypothesis that linear protein sequences from known human viral and bacterial pathogens could be useful in predicting potential autoepitopes on human CNTNAP2 we screened for CSSRs by comparison of human and mouse CNTNAP2 proteins against known bacterial and viral protein databases using NCBI Protein-BLAST [25] (Table 1). The candidate CSSR had to be at least 5 aa in length, within the predicted extracellular region of both human and murine CNTNAP2, be within a predicted B-cell epitope [26–28], and be from a known human pathogen. The final 8 aa peptides containing CSSRs were lastly selected *a priori* for further evaluation (Table 2).

### 3.2 CNTNAP2-binding Antibodies in Sera from Children with Autism and Non-autistic Controls

**Fig. 1:**
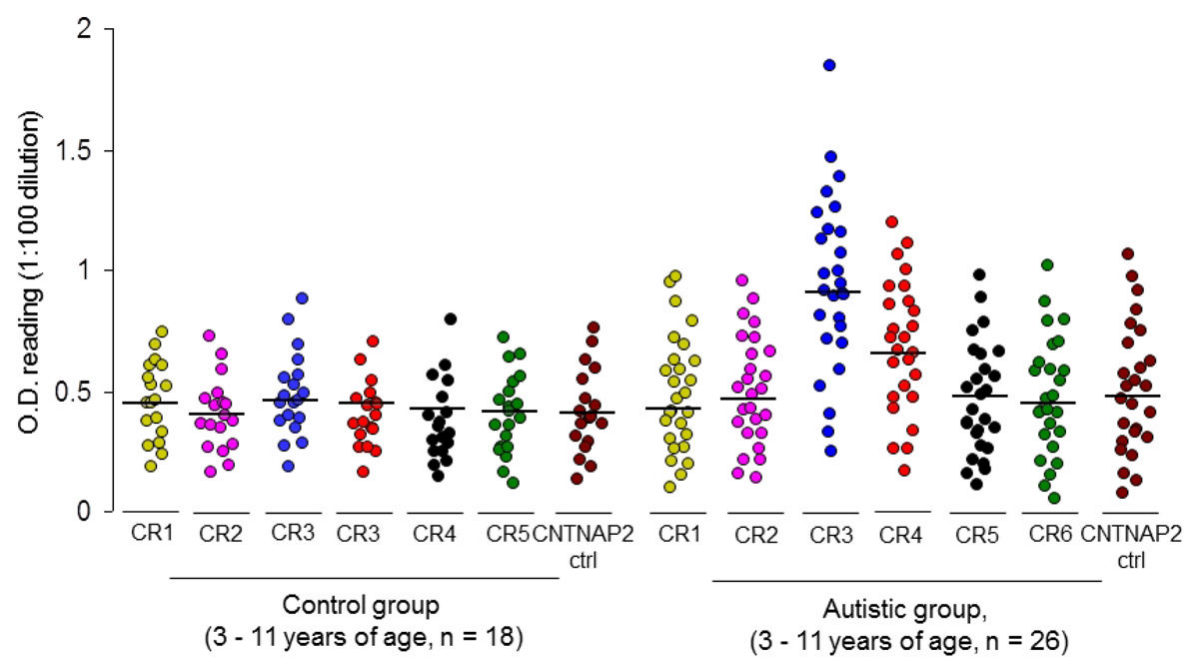
CNTNAP2-binding antibodies in sera from children with autism and non-autistic controls. Levels of serum antibodies binding to 8 aa CNTNAP2 autoantibody detection peptides containing analogous CSSR sequence from corresponding pathogen proteins (Table 2) were screened by ELISA. Each dot represents a mean optical density reading (O. D.; 450 nm; 1:100 dilution) for each individual (n = 26 for autistic children; n = 18 for non-autistic controls) for a respective level of serum antibodies binding to a given CNTNAP2 autoantibody detection peptide (CR). Levels of CSSR3 (CR3) and CSSR4 (CR4) autoantibody titers were significantly elevated in children with autism compared with non-autistic control sera (P < .05). There were no significant differences in autoantibody titer binding to other CNTNAP2 autoantibody detection peptides compared to CNTNAP2 control peptide (P > .05)

Sera from children 3–11 years of age with autistic disorder (n = 26), and non-autistic controls (n = 18), were obtained (Table 3) and screened by ELISA for the presence of antibodies against 8 aa peptide targets of CNTNAP2 (Table 2) containing sequence-similarity with proteins from known human pathogens. Compared with the CNTNAP2 control peptide target, significant elevations in antibody binding were only observed to CSSR3 and CSSR4 in those with autism (Fig. 1). Although pathogen exposure profiles of the individuals are unknown and the groups are characteristically dissimilar (Table 3) these observations suggested that some children have circulating antibodies able to bind regions of CNTNAP2 that are sequence-similar to proteins from known human pathogens.

### 3.3 CNTNAP2 Binding Antibodies Generated in Mice Pre-injected with LPS and Immunized with a Pathogen Peptide Containing the CSSR

**Fig. 2:**
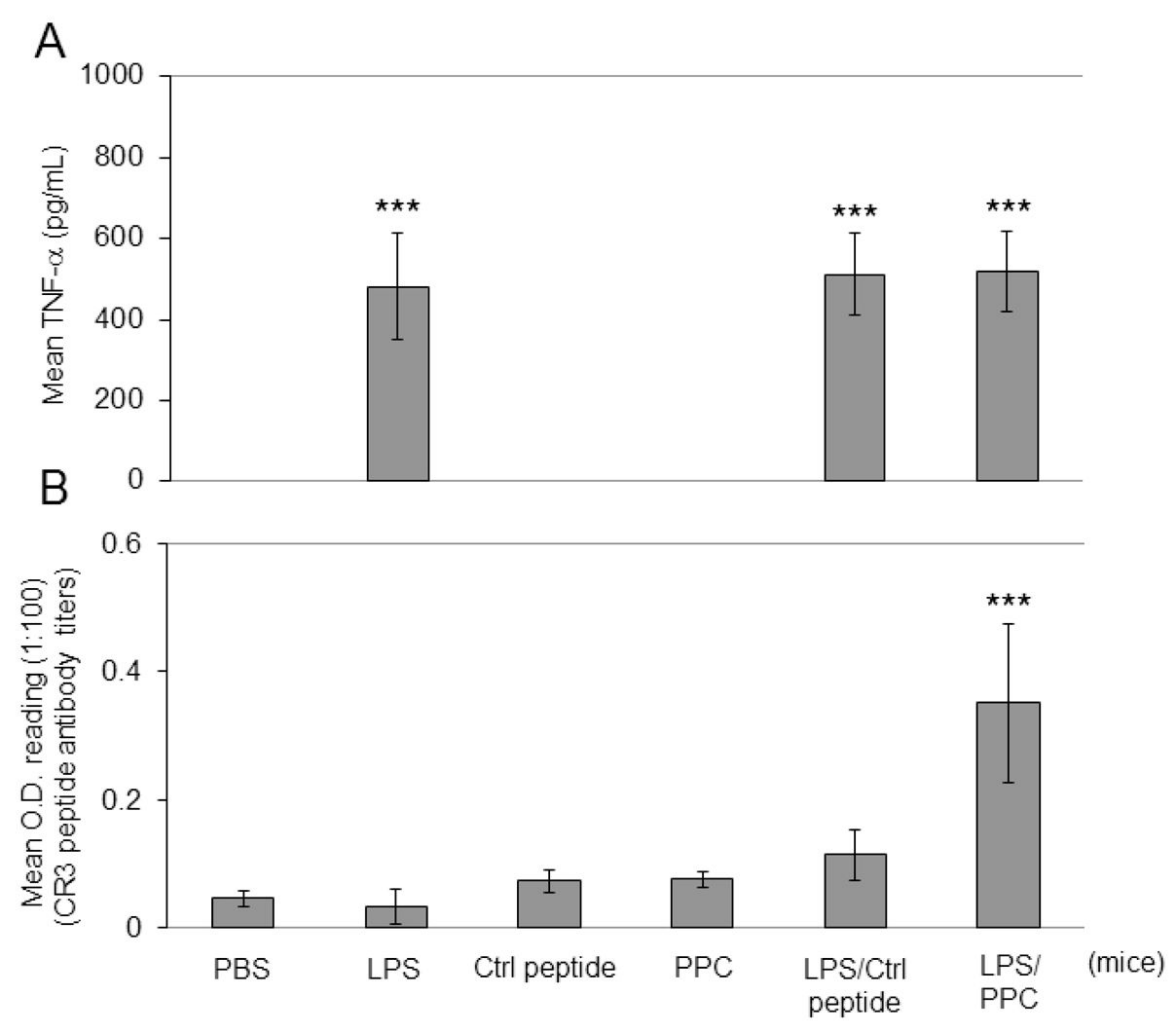
CNTNAP2 binding antibodies generated in mice pre-injected with LPS and immunized with PPC. Wild-type C56BL/6 (WT) mice (4 week old, n = 8, 4♀/4♂ per group) intraperitoneally (i.p.) injected with PBS or LPS (10 μg/mouse) with and without control peptide (Ctrl) or PPC (200 μg/mouse) immunizations. The same procedure was repeated one week later. ELISA for TNFα (A) and CSSR3 binding antibody titer (B) were determined 3 weeks after last immunization. The results are presented as mean ± SD of TNFα (pg/mL) for (A) and mean ± SD of O.D. reading at 1:100 dilution for (B). ***P <.001

Next, given that some children displayed elevations in serum antibody binding to its target sequence CSSR3 was selected for functional characterization in a mouse model of acute infection. Four-week-old mice C57BL/6 mice were subjected to PBS or LPS pre-treatment (10 μg/mouse) 2 days prior to immunization with a 20 aa peptide from pathogen peptide containing the CSSR (PPC) or control peptide (a portion of CNTNAP2 found not to have significant linear protein sequence similarity to known human bacterial or viral pathogen proteins). The same procedure was repeated one week later and mice were sacrificed after motor function testing; at approximately 8 weeks. Only those mice treated with both LPS pre-treatment and PPC expressed significantly elevated levels of antibodies able to bind the CSSR3 peptide (Fig. 2B) by ELISA. This suggested that in mice a peptide derived from a pathogen protein with a CSSR could induce the generation of antibodies binding the analogous region of CNTNAP2 with LPS pretreatment. As expected, LPS pre-treatment was associated with serum TNFα elevations (Fig. 2A).

### 3.4 CSSR3 Peptide Binding Antibodies Injure Neuronal Cells

**Fig. 3:**
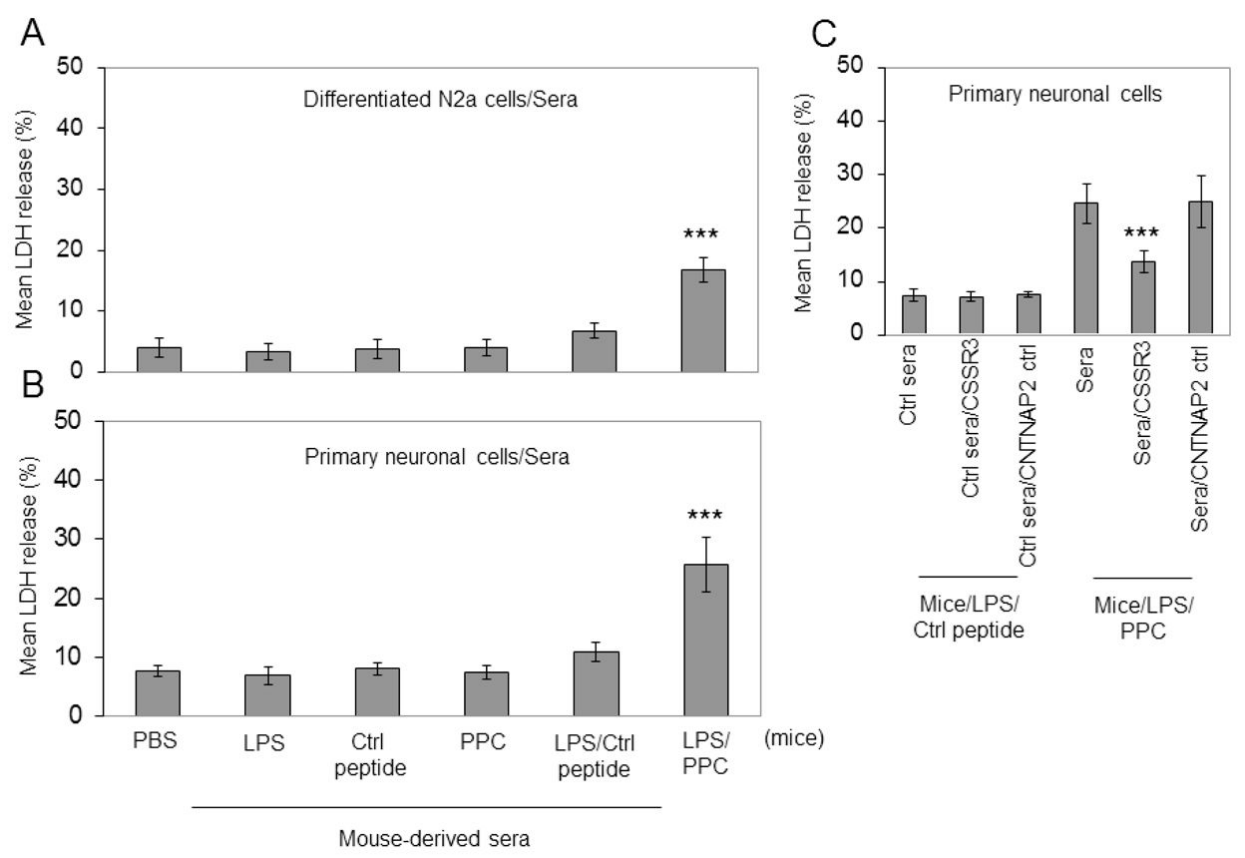
CSSR3 peptide binding antibodies injure neuronal cells. Pooled sera from control and PPC immunized groups were collected 3 weeks after last immunization (8 week old, n = 8, 4♀/4♂ per group) and used to treat differentiated N2a cells (A) and mouse primary neuronal cells (B) for 24 h and analyzed by LDH assay. Data are presented as mean ± SD of LDH release (%) from each incubated group normalized by total cellular protein. (C) Mouse primary neuronal cells incubated with sera from “control mice” (immunized with Ctrl peptide + LPS pre-injection) or “PPC mice” (immunized with PPC + LPS pre-injection) for 24 hours with and without pre-incubation with CSSR3 or CNTNAP2 ctrl peptides (5 μg/mL) for 1 h at 37°C. Ctrl sera, the pooled serum from “control mice.” Ctrl sera/CSSR3, pooled serum from “control mice” pre-incubated with CSSR3 peptide. Ctrl sera/CNTNAP2 ctrl, pooled serum from “control mice” pre-incubated with CNTNAP2 ctrl peptide. Sera, the pooled serum from the PPC mice. Sera/CSSR3, the pooled serum from the PPC mice pre-incubated with CSSR3 peptide. Sera/CNTNAP2 ctrl, the pooled serum from the PPC mice pre-incubated with CNTNAP2 ctrl peptide

To further characterize the functional effects of CSSR3 binding, the pooled sera from the same mice were further analyzed through incubation with neuronal cells to monitor cell death by LDH release over 24 h. Sera from the PPC treated group pre-treated with LPS, but not sera from the group treated with PPC alone or control peptide with or without LPS-pre-treatment, displayed significant elevations in LDH release by differentiated neuron-like N2a cells (Fig. 3A) and murine primary culture neurons (Fig. 3B).

The LDH release of the sera from mice subjected to PPC immunization and LPS pre-treatment could be mitigated by pre-mixing the sera, prior to incubation with neuronal cells, with the CSSR3 peptide but not the CNTNAP2 ctrl peptide (Fig. 3C). These observations suggested that CSSR3 binding antibodies produced in mice pre-treated with LPS displayed neurotoxic properties dependent on their ability to bind a specific extracellular region of murine CNTNAP2 (545–550 aa).

### 3.5 Elevations in CD3+ Cells in Brains of Mice Treated with LPS and PPC

**Fig. 4:**
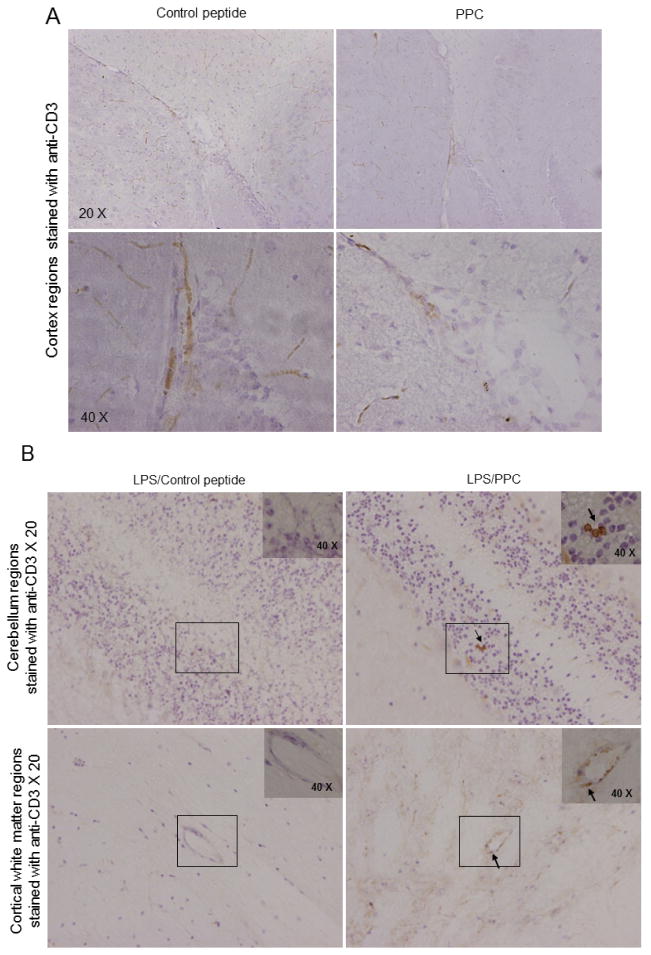
Elevations in CD3+ cells in brains of mice treated with LPS and PPC. (A) Representative gray matter cortical regions from mice immunized with control peptide (Ctrl) or PPC peptide with pre-treatment with LPS (8 week old, n = 8, 4♀/4♂ per group). Significant CD3+ T-cell infiltration was not observed in the areas of cortical gray matter analyzed in either group. (B) Representative cortical cerebellar and cortical white matter regions from mice immunized with Ctrl peptide or PPC with LPS pre-treatment. Mice that received LPS pre-injection and were immunized with PPC peptide displayed CD3+ T-cell infiltration in the cerebellum and cortical white matter. Mice immunized with Ctrl peptide + LPS pre-injection did not display CD3+ T-cell infiltration in these regions

Several brain regions were evaluated by immunohistochemistry (IHC) for CD3+ immunoreactivity. Although no differences were observed between the groups in the analyzed regions of cortical gray matter (Fig. 4A), mice subjected to LPS pre-treatment and immunization with PPC displayed increased cortical white matter as well as cerebellar CD3+ (Fig. 4B) and CD4+ immunoreactivity (data not shown); whereas other groups displayed predominately blood vessel associated T-cell CD3+ and CD4+ immunoreactivity.

### 3.6 IFNγ and TNFα Elevations in Brains of Mice Treated with LPS and PPC

**Fig. 5:**
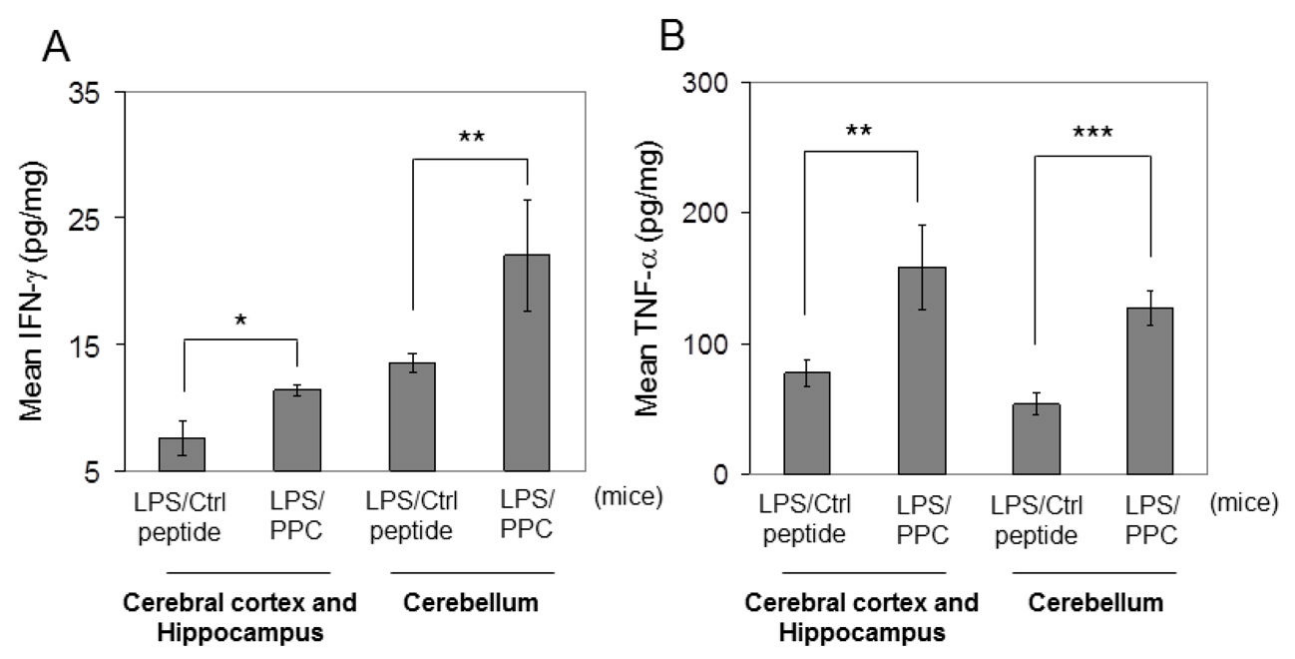
IFNγ and TNFα elevations in brains of mice treated with LPS and PPC. Mouse brain tissue homogenates (8 week old, n = 8, 4♀/4♂ per group) were evaluated for IFNγ (A) and TNFα (B) by ELISA. The results are presented as mean ± SD of brain IFNγ or TNFα (pg/mg total protein). Both cytokines were not detectable in brain tissues from other control groups (data not shown). *P< .05; **P< .005; **P< .001

Furthermore, dramatic elevations in central nervous system (CNS) levels of IFNγ (Fig. 5A) and TNFα (Fig. 5B) cytokines were observed in the LPS pre-treatment and PPC group compared with the LPS pre-treatment/control peptide group.

### 3.7 LPS and PPC Treatment Results in Abnormal Motor Function in Mice

**Fig. 6:**
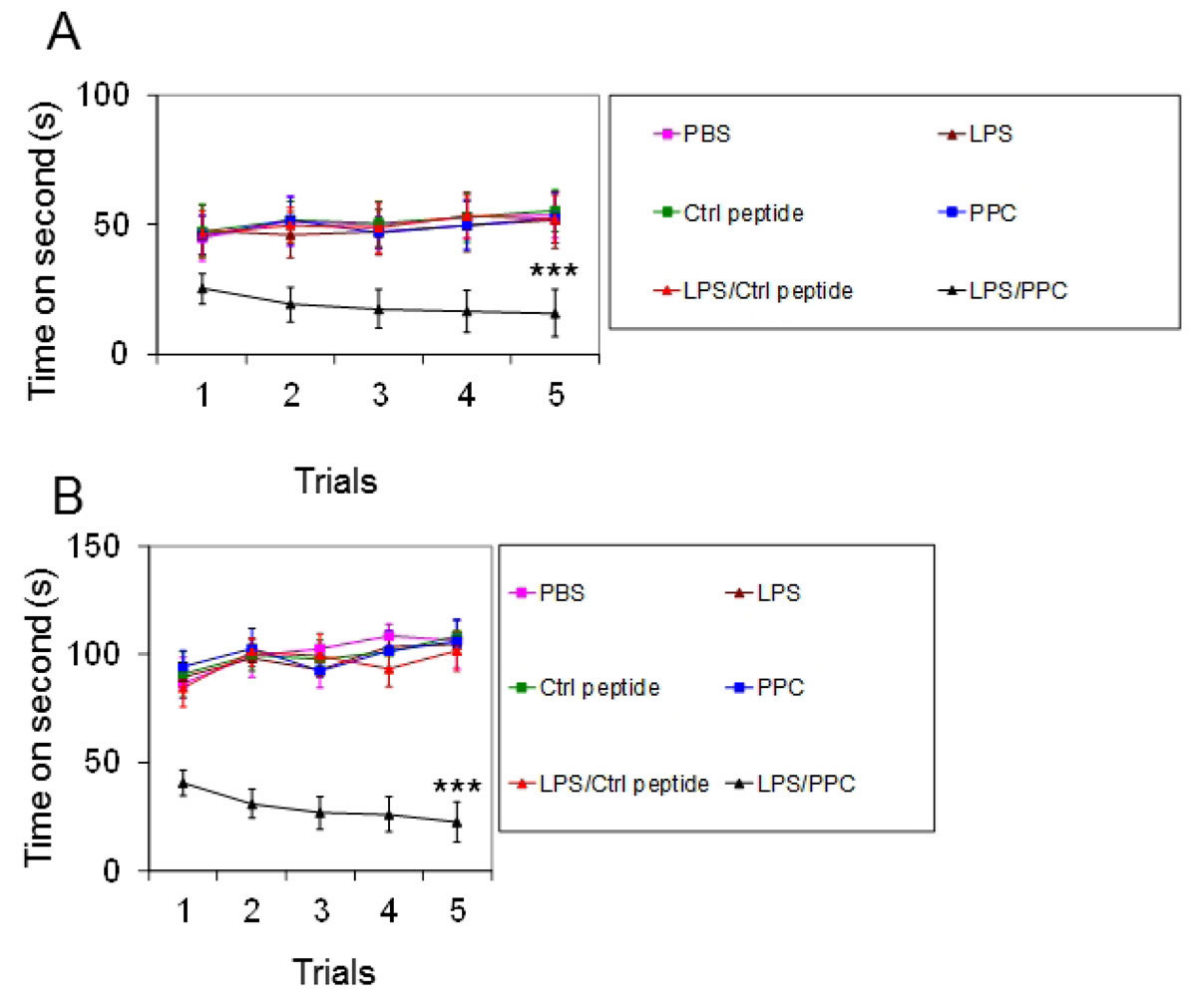
LPS and PPC treatment results in abnormal motor function in mice. Wild-type C56BL/6 mice were immunized with PPC or control peptide (Ctrl) with LPS or PBS pre-treatment and subjected motor function testing (8 week old, n = 8, 4♀/4♂ per group). Motor balance analysis was conducted using RotoRod. (A) Time before falling from a stationary RotoRod. (B) Time before falling off an accelerating RotoRod. Results are presented as mean ± SD from five trials. ***P <.001

Observations of elevations in CNTNAP2 autoantibodies have been made in some patients [13]; although often associated with muscular hyperactivity, including spasm, rigidity, and myotonia, fatigue and exercise intolerance are also frequently observed. Thus, the same C57BL/6 mice, 3 weeks after last treatment, prior to sacrifice, were evaluated for fatigue and exercise intolerance by RotoRod analysis. Mice in the LPS pre-treatment, PPC immunization group displayed significantly greater fatigue and exercise intolerance during RotoRod analysis including shorter times before falling from a stationary RotoRod (Fig. 6A) and shorter times before falling from an accelerating RotoRod (Fig. 6B) compared with other groups evaluated.

Autoantibodies observed in cases of autoimmune encephalitis and/or neuromyotonia have not been shown to involve binding to the region of CNTNAP2 characterized here (545–550 aa) and to our knowledge this is the first study to screen for potential autoantigenic regions of CNTNAP2 using the methods presented here. Known to be associated with immune system dysregulation [8–11,22,32–34], subsets of children with autism tended to display elevations of serum antibody binding to CSSR3 and CSSR4 (Fig. 1) compared with non-autistic children; however these data are hampered by the dissimilarity of non-CNTNAP2 binding antibody variables between the two groups evaluated (Table 3) and the unknown or incomplete medical histories of the patients.

Despite the relatively small region of similarity the 6 aa long CSSR of the PPC fulfills known requirements for antigen function [35–37]. The PPC was used to immunize mice pre-treated with LPS to further characterize the autoantigenic potential of a small segment (545–550 aa) of the extracellular region of human and murine CNTNAP2. This treatment was associated with elevations in antibodies able to bind the analogous region of CNTNAP2 (Fig. 2), CD3+ and CD4+ (data not shown) cells and inflammatory cytokines in CNS tissues (Fig. 4, 5) and motor dysfunction (Fig. 6); only in the presence of LPS pre-treatment. LPS and PPC treated mice showed signs of an encephalitis-like reaction with increased parenchymal cortical white matter as well as cerebellar CD3+ (Fig. 4B) and CD4+ immunoreactivity (data not shown); whereas without the combination of LPS and PPC the pattern was that of blood vessel associated T-cell CD3+ and CD4+ immunoreactivity; similar to untreated controls. These data together suggest that only in the presence of strong inflammatory responses, mimicking acute gram-negative bacterial infection driven by LPS, was immune tolerance to the CNTNAP2 self-antigen able to be broken.

Whereas in 4–8 week old mice treatment with LPS and PPC lead to encephalitis-like responses and motor dysfunction, it is tempting to speculate whether earlier exposure, perhaps *in utero*, would lead to more profound immune and nervous system dysfunction and abnormal development. In light of the key role of CNTNAP2 in neuronal activation, migration and neural-glial interactions [18,19,38] further studies are needed to determine whether anti-CNTNAP2-mediated encephalopathy, if occurring during critical widows of brain development, could represent a significant risk factor for abnormal neurodevelopment. Prior studies present compelling evidence of a role for the immune system in the pathogenesis of subsets of neurodevelopmental disorders [8–12,22,23,32–34].

Importantly, the results have significant limitations including that a synthetic linear peptide representing a small fragment of CNTNAP2, not in its native form, was used to immunize and evaluate the effects of the CSSR in mice and detect CSSR-binding antibodies in human samples. Although the antibodies binding to CSSR3 generated by LPS and PPC pretreatment appeared to bind CNTNAP2 in its native form on neuronal cells (Fig. 3), it remains to be determined whether the extracellular region of (545–550aa) CNTNAP2 analogous to the PPC would be available for antibody binding in its native conformation in humans. Further, the human sample data contained within the present study is significantly limited by the small sample size and dissimilarity between groups as well as the lack of or incomplete medical histories. Further complete immunological characterization of the evaluated mice was not completed prior or after LPS and PPC treatment.

## 4. CONCLUSION

CNTNAP2 contains a potential autoepitope within the extracellular region.

## 5. FUTURE DIRECTIONS

Molecular mimicry is implicated in neurological disorders associated with anti-CNTNAP2 antibodies. Analysis of the relative affinity of antibodies from patients with autistic disorder binding to the 545–550 aa region of CNTNAP2 against antisera from mice immunized using the same region as well as analysis of antibody affinity-to-neurotoxicity relationships and visualized regional binding characteristics on human neurons could support the hypotheses that antibodies binding to the 545–550 aa region of CNTNAP2 are causative in human neurological disorders.

## Figures and Tables

**Table 1:** CSSRs from proteins from human viral and bacterial pathogens

Protein	Pathogen Sequence	Human CNTNAP2
[YP_006827301.1] ABC transporter, Bacillus thuringiensis	105 LKLDHYP 111	1127 LKLDHYP 1133
[WP_006006950.1] aminotransferase, Desulfovibriopiger	138 PWHLD 142	1230 PWHLD 1234
[YP_005986680.1] beta-N-acetylhexosaminidase, Propionibacterium acnes	775 ARYVR 779	156 ARYVR 160
[WP_005989682.1] DNA/RNA helicase, Desulfovibrioafricanus	79YYCNCD 85	800 YYCNCD 805
[NP_880571.1] filamentous hemagglutinin/adhesin, Bordetella pertussis	205 GRIGL 209	170 GRIGL 174
[NP_880571.1] filamentous hemagglutinin/adhesin, Bordetella pertussis	8 LVFSH 12	410 LVFSH 414
[NP_880571.1] filamentous hemagglutinin/adhesin, Bordetella pertussis	3041 ANVSID 3046	545 ANVSID 550
[ABX58360.1] hemagglutinin, Influenza A virus	490 GCMES 494	335 GCMES 339
[ADI46314.1] hemagglutinin, Influenza A virus	493 GCMESI 498	335 GCMESI 340
[WP_002570578.1] hypothetical protein, Clostridium bolteae	919 YSQLF 923	220 YSQLF 224
[WP_004614047.1] hypothetical protein, Clostridium nexile*	1308 ARYVR 1312	156 ARYVR 160
[WP_010496242.1] hypothetical protein, Paenibacilluselgii*	1072 ARYVRI 1077	156 ARYVRI 161
[AAF85674.1] large protein, Measles virus	91 NQDLF 95	391 NQDLF 395
[AAL83746.1] large protein, Mumps virus	2004 SSFTT 2008	1068 SSFTT 1072
[WP_001175565.1] NADH oxidase, Bacillus cereus	105 LKLDHYP 111	1127 LKLDHYP 1133
[O40955] non-structural polyprotein p200, Rubella virus	214 WHLDH 218	1231 WHLDH 1235
[AAG31334.1] ORF 169b, Lactobacillus phage mv4*	125 FLKLD 129	1125 FLKLD 1129
[WP_006763014.1] outer membrane protein, Burkholderia dolosa	1134 VSYHLP 1139	1134 VSYHLP 1139
[WP_002571612.1] polysaccharide deacetylase, Clostridium bolteae	421 PYHQD 425	126 PYHQD 130
[ABV01075.1] polymerase PB1, Influenza A virus	278 KKAKL 282	241 KKAKL 245
[YP_005052428.1] SNF2-related protein, Desulfovibrio africanus	131 YYCNCD 136	800 YYCNCD 805

Peptide sequences are identified by National Center for Biotechnology Information (NCBI) or GenBank accession numbers.

* Not recognized as direct human pathogens.

**Table 2:** CNTNAP2 Autoantibody Detection Peptides

Pathogen NCBI accession #, peptide name	Peptide sequence	Human CNTNAP2 location
[NP_880571.1], CSSR1	GEGRIGLR	CNTNAP2-170
[NP_880571.1], CSSR2	GLLVFSHF	CNTNAP2-410
[NP_880571.1], CSSR3	FANVSIDM	CNTNAP2-545
[AAF85674.1], CSSR4	RLNQDLFS	CNTNAP2-391
[AAL83746.1], CSSR5	YISSFTTD	CNTNAP2-1068
[O40955], CSSR6	DPWHLDHL	CNTNAP2-1231
[NP_054860.1], CNTNAP2 Control	SGLPHVAF	CNTNAP2-41

**Table 3:** Characteristics of control and autistic children

	Controls (n=18)	Autism (n=26)	p value
Female (%)	44.44	15.39	p<.01
Age (years)	5.71±1.42	4.00±1.366	p<.001

## ABBREVIATIONS

ADI-R: Autism Diagnostic Interview, Revised
ANOVA: Analysis of variance
BLAST: The Basic Local Alignment Search Tool
Caspr2: contactin-associated protein-like 2
CD3: cluster of differentiation 3
CD4: cluster of differentiation 4
CNS: central nervous system
CNTNAP2: contactin associated protein-like 2
CSSR: CNTNAP2 sequence similar region
DAB: 3,3′-diaminobenzidine
EDTA: Ethylenediaminetetraacetic acid
h: hour(s)
IFNγ: interferon-gamma
IgG: immunoglobulin G
i.p: intraperitoneal
LDH: lactose dehydrogenase
LPS: lipopolysaccharide
M: molarity
N: Normality
NCBI: National Center for Biotechnology Information
PBS: phosphate buffered saline
PMSF: phenylmethylsulfonyl fluoride
PPC: pathogen peptide containing CSSR
TNFα: tumor necrosis factor-alpha
